# Assessment of the dimensionality of the Wijma delivery expectancy/experience questionnaire using factor analysis and Rasch analysis

**DOI:** 10.1186/s12884-016-1157-8

**Published:** 2016-11-21

**Authors:** J. F. Pallant, H. M. Haines, P. Green, J. Toohill, J. Gamble, D. K. Creedy, J. Fenwick

**Affiliations:** 1 Department of Rural Health, The University of Melbourne, Graham St, Shepparton, Australia; 2 Faculty of Health Sciences and Medicine, Bond University, Queensland, Australia; 3 Menzies Health Institute, Griffith University, Meadowbrook Queensland, Australia; 4 Gold Coast University Hospital Southport, Queensland, 4215 Australia

**Keywords:** Fear of childbirth, Measurement, Factor analysis, Rasch analysis

## Abstract

**Background:**

Fear of childbirth has negative consequences for a woman's physical and emotional wellbeing. The most commonly used measurement tool for childbirth fear is the Wijma Delivery Expectancy Questionnaire (WDEQ-A). Although originally conceptualized as unidimensional, subsequent investigations have suggested it is multidimensional. This study aimed to undertake a detailed psychometric assessment of the WDEQ-A; exploring the dimensionality and identifying possible subscales that may have clinical and research utility.

**Methods:**

WDEQ-A was administered to a sample of 1410 Australian women in mid-pregnancy. The dimensionality of WDEQ-A was explored using exploratory (EFA) and confirmatory factor analysis (CFA), and Rasch analysis.

**Results:**

EFA identified a four factor solution. CFA failed to support the unidimensional structure of the original WDEQ-A, but confirmed the four factor solution identified by EFA. Rasch analysis was used to refine the four subscales (Negative emotions: five items; Lack of positive emotions: five items; Social isolation: four items; Moment of birth: three items). Each WDEQ-A Revised subscale showed good fit to the Rasch model and adequate internal consistency reliability. The correlation between Negative emotions and Lack of positive emotions was strong, however Moment of birth and Social isolation showed much lower intercorrelations, suggesting they should not be added to create a total score.

**Conclusion:**

This study supports the findings of other investigations that suggest the WDEQ-A is multidimensional and should not be used in its original form. The WDEQ-A Revised may provide researchers with a more refined, psychometrically sound tool to explore the differential impact of aspects of childbirth fear.

**Electronic supplementary material:**

The online version of this article (doi:10.1186/s12884-016-1157-8) contains supplementary material, which is available to authorized users.

## Background

Fear of childbirth has been linked with a number of negative consequences for a woman’s physical and emotional wellbeing. These include pregnancy complications, increased length of labour [1], use of anaesthesia during labour [2, 3], and increased risk of caesarean section deliveries [4, 5]. The content of a woman’s fear may include feelings of lack of control, fear of pain, fear of humiliation, fear for the life and wellbeing of her baby, fear for her own life, and fear of perineal tearing [6]. Many women are fearful of repeating a previous negative birthing experience [7].

One of the most commonly used tools for the measurement of fear of childbirth is the Wijma Delivery Expectancy/Experience Questionnaire (WDEQ-A) which was developed to “measure fear of childbirth by means of the woman’s cognitive appraisal regarding the delivery” [8] (p. 85). Since its development the WDEQ-A has been translated into a number of languages, and has been used in a wide range of studies exploring the correlates and consequences of elevated levels of fear [9–13]. Although the 33-item WDEQ-A was originally conceptualized as a unidimensional measure, subsequent investigations by other researchers using factor analysis have suggested that it may in fact be multidimensional, tapping a number of different aspects [9, 10, 14, 15]. Johnson and Slade [10] conducted the first factor analysis of the WDEQ-A and concluded that ‘it measured four clear dimensions that are conceptually distinct’ (p.1220). To achieve a satisfactory solution the authors of that study found it was necessary to remove three items (items 26: ‘let happen’, and 28:‘funny’ and item 30: ‘obvious’), with the final four dimensions labeled *Fear, Lack of positive anticipation, Isolation* and *Riskiness*.

Australian researchers [14] conducted similar analyses. The four factors defined as ‘fear’, ‘isolation’, ‘lack of positive anticipation’ and ‘riskiness’ in the UK study were similarly identified in the Australian sample, however there were several differences in the actual items included in each factor see Fenwick et al. [14] page 673.

Subsequent analysis undertaken in Norway [16], using both exploratory and confirmatory factor analysis, suggested that a six-factor model showed the best fit, after the removal of eight items. The final solution for the remaining 25-item version of the WDEQ-A included subscales labelled *Fear, Negative Appraisal, Loneliness, Lack of self-efficacy, Lack of positive anticipation* and *Concerns for the child*. This six factor structure was later confirmed in a large multi-country European study [12] although no items were removed as none had communalities less than 0.3 [12]. The authors decided that essentially the same factors were found in each of the six European countries and maintained the same factor names as the earlier Norwegian study [16] however these factors were not made up of all the same original items in all the countries [12].

In a study validating an Italian version of the WDEQ-A, [9] the authors raised a number of concerns about the factor analysis procedures undertaken in earlier reports. In particular they noted that in the four factor solutions reported in the literature, items were retained in the scale despite low communalities, failure to load substantially on a single factor, or cross loadings. They also noted that previous researchers had not formally tested the factor structure after removing items from the scale, an important step in determining the factor structure of a scale. Fenaroli and Saita [9] also questioned the validity of retaining a subscale containing only two substantially loading items, labelled *Riskiness* by Johnson and Slade [10], and Fenwick et al. [14], and *Concerns for the child* by Lukasse et al. [12] and by Garthus-Niegal et al. [16]. Although these items may conceptually be related to the underlying concept of fear of childbirth, the two items are not sufficient, psychometrically, to form a robust subscale [9]. This concern about the robustness of the two-item *Riskiness* factor was also raised in a Japanese validation study of the WDEQ-A [15], suggesting that the factor may be “weak and unstable” (p.331). These authors drew attention to the practice of retaining items with relatively low factor loadings and suggested that “more careful attention to the items will be needed in future research” (p.331).

Although many researchers using the WDEQ-A over the past 15 years have calculated a single total score [3, 10, 17] this requires the assumption that the scale is unidimensional. However all authors to date that have tested the dimensionality of the scale have identified distinct factors (between four and six) suggesting multidimensionality [9, 10, 14]. Studies using confirmatory factor analysis have formally tested the appropriateness of a single factor solution and reported very poor fit statistics [9, 16]. Both studies reported comparative fit index (CFI) values below .6, well below the accepted guidelines of .95 for good model fit and .90 for moderate fit [18]. These results, suggesting that the WDEQ-A items do not measure a single underlying dimension, are supported by the low correlations among the factors reported by some authors. Garthus-Niegel et al. for example, reported correlations between the *Concerns for the child* factor and other WDEQ-A factors ranging from a high of only .298 and a low of .145 [16]. Values this low suggests that this set of items share less than 9% variance with the other factors identified in the WDEQ-A. A summary of studies which have reported results of factor analysis of the WDEQ-A can be found in Table 1.

**Table 1:** Summary of factor analysis of WDEQ

Article	Language	Type of analysis	Number of factors	Number of items	Factor labels
Johnson & Slade [10]	English	EFA	4	30	Fear, Isolation, Lack of positive anticipation, Riskiness
Fenwick et al. [14]	English	EFA	4	33	Fear, Isolation, Lack of positive anticipation, Riskiness
Garthus-Niegel et al. [16]		CFA	6	25	Fear, Negative appraisal, Loneliness, Lack of self efficacy, Lack of positive anticipation, Concerns for the child
Takegata et al., [16]	Japanese	EFA	4	33	Fear, Lack of positive anticipation, Isolation, Riskiness
Fenaroli et al., [9]	Italian	EFA	4	16	Fear, Negative feelings, Lack of confidence, Negative thoughts
Fenaroli et al., [9]	Italian	CFA	3	14	Fear, Negative feelings, Lack of confidence
Lukasse et al. [12]	Norwegian Swedish Danish Estonian Flemish Icelandic Russian	EFA	6	33	Fear, Negative appraisal, Loneliness, Lack of self efficacy, Lack of positive anticipation, Concerns for the child

Low correlations among the factors indicate that women with high scores on one factor do not necessarily have high scores on other factors. For example, just because a woman who feels concern that their child would die or be injured during the labour/birth (item 32, 33) does not necessarily mean that they will feel “lonely” (item 3) or “abandoned” (item 15). The combination of these items to form a single score is clearly inappropriate and may result in the loss of potential information for clinicians in particular. A profile, providing separate subscale scores representing each factor, may prove to be more useful in planning an intervention or providing customized support for an individual woman.

In order to identify and extract a set of subscales from the WDEQ-A that can be used by future researchers and clinicians it is important that the items be subjected to rigorous testing using the latest in psychometric procedures. The importance of good psychometric procedures was emphasized in a recent edition of Journal of Reproductive and Infant Psychology which was devoted to the topic of measurement of psychological health in the prenatal period [19]. These authors suggest that ‘there is much to be gained from new statistical techniques and approaches when developing measures to capture the complexity of psychological health in the perinatal period’ [19] (p. 436). They went on to highlight that: ‘whatever we measure requires rigorous and robust evaluation of the measure both in terms of psychometric standards and interpretation of those standards’ [19] (p.437).

Although the WDEQ-A has been analysed using a number of classical test theory approaches (exploratory factor analysis, confirmatory factor analysis), to date the scale has not yet been subjected to Rasch Analysis, which is based on modern test theory. Rasch analysis is a procedure increasingly being used within the health sciences, medical, psychology and business literatures. It has been used to psychometrically assess many hundreds of clinical tools [20] including the Perinatal Attachment Index [21] and assessment of the birth experience [22]. For a review of the use of Rasch analysis in nursing research, see Hagquist et al. [23]. Unlike classical test theory approaches, Rasch analysis involves a full assessment of all aspects of a scale’s functioning, including its response format, item fit, potential bias, suitability for particular groups, dimensionality and targeting. It allows scales to be refined by removing items that do not fit with the underlying dimension being measured. This assists in the development of short, concise scales that are unidimensional, and are free from item bias.

The use of Rasch analysis to identify and remove WDEQ-A items that do not directly tap the underlying dimension may serve to increase its potential clinical application. In its current form of 33 items the WDEQ-A has been criticized as being too long and complex to be used routinely in clinical settings and to be accurately translated into multiple languages [24]. A recent qualitative study from the United States challenged the utility and appropriateness of the WDEQ-A in its current form for use as a screening tool in a U.S. context where there are many systematic differences in healthcare compared to the Swedish context where the questionnaire was originally developed [25]. In response to this criticism a number of articles have been published recently proposing alternative short measures of childbirth fear [26, 27]. The multidimensional nature of the WDEQ-A, suggested by recent factor analytic studies, may be able to be used to advantage in developing a set of subscales, measuring additional aspects of the construct, over and above the original focus on fear of childbirth.

The aim of this study was to undertake a very detailed psychometric assessment of the WDEQ-A using techniques from both classical test theory (EFA, CFA) and modern test theory (Rasch analysis). The goal was to explore the dimensionality of the scale using EFA and CFA and to identify possible subscales of the WDEQ-A that may have clinical and research utility. Rasch analysis was used to formally assess the response format, suitability of the items, item bias, internal consistency reliability, dimensionality and targeting of the subscales. Additional analyses were also undertaken to explore the correlations among the WDEQ-A subscales and their association with other existing measures, and selected demographic and obstetric characteristics.

## Method

This study involved secondary analysis of data from a large Australian randomised control trial designed to test the effectiveness of a midwife led psycho-education intervention to reduce childbirth fear – The BELIEF study [28].

### Participants

Two thousand, three hundred and eleven pregnant women from antenatal clinics in Queensland Australia were invited to participate. Of these 61% (*n* = 1410) were recruited [13]. The data used for the current study are from time point one administered to women in their second trimester. The results of the BELIEF study are reported elsewhere [13].

### Procedure

The participants completed three self-report questionnaires during their second trimester, 36 weeks of pregnancy, and 4–6 weeks after birth.

### Measures

The questionnaires included two instruments for measurement of fear of birth: the Wijma Delivery Expectancy/Experience Questionnaire (WDEQ-A) [8] and FOBS- The Fear of Birth Scale^TM^ [26]; the Edinburgh Postnatal Depression Scale (EPDS) in addition to socio-demographic, medical and psychosocial questions.

The WDEQ-A is a 33-item, 6-point Likert scale questionnaire [8]. Items refer to expectations and experiences before birth, each scoring from 0 to 6. Women have to answer while imagining how labour and delivery are going to be, and how they expect to feel. Items with positively formulated questions are reverse-scored. The sum score ranges from 0 to 165; the higher the score is, the greater the fear of childbirth.

FOBS - The Fear of Birth Scale^TM^ [26] is a two-item visual analogue scale that includes the constructs of worry and fear. It consists of the question “How do you feel right now about the approaching birth,” with the anchor words, calm/worried on one 100 mm scale and no fear/strong fear on the other 100 mm scale. The two values are then averaged to one score with the higher the score the stronger the fear. The FOBS^TM^ has been used in studies in Australia [26] and Sweden where it was translated into multiple languages [24].

The EPDS is a 10 item self-report questionnaire designed to screen for depression. Range of scores is from 0 to 30 with postnatal scores above 12 indicative of probable depression [29]. Questions 3 and 5–10 are scored in reverse, with the top response scored as a three and the bottom response scored as 0. For additional analysis this study also used the components of the EPDS which specifically measures anxiety - EPDS-3A (items 3, 4, and 5) [30].

### Statistical analyses

A two-step process was undertaken to explore the factor structure of the WDEQ-A using the procedure suggested by Gerbing and Hamilton [31]. The total datafile (*n* = 1410) was randomly divided into two files, one used for exploratory factor analysis (EFA: *n* = 683) and the other retained for the later confirmatory factor analysis (CFA: *n* = 727). The size of both datafiles well exceeded the recommended sample size for EFA and CFA [32].

EFA on Sample 1 was undertaken using SPSS Version 22. The suitability of the data was confirmed using the Kaiser-Meyer-Olkin measure of sampling adequacy (values above .6 considered acceptable [33]), and a significant Bartlett’s test of sphericity [34]. Principal components analysis was used to extract the factors, and oblimin rotation was used to improve the interpretability of the solution. To identify the number of factors to retain three decision rules were utilized: Kaiser’s criterion, retention of eigenvalues above 1; Catell’s screeplot [35] and Horn’s parallel analysis [36]. This involved the comparison of eigenvalues from the PCA with a set derived from a randomly generated datafile [37] with the same number of items and cases. Components with eigenvalues exceeding those obtained from the random datafile were retained.

Using a separate datafile (Sample 2: *n* = 727) Confirmatory Factor Analysis (CFA) was conducted using MPlus Version 7.1 [38]. Weighted least squares means and variance adjusted estimation [39] was used given the ordinal nature of the data [40]. A range of fit statistics was used to assess overall model fit. The chi square statistic and associated *p* value is very sensitive to sample size therefore a number of other adjusted fit statistics are typically reported [18]. Two incremental fit statistics were reported (comparative fit index: CFI; goodness of fit index GFI), with values less than 0.90 indicating lack of fit, values between 0.90 and 0.95 indicating reasonable fit and values above .95 representing good fit. The root mean-square error of approximation (RMSEA), which Byrne (2010) [18] suggests is the most informative fit statistic, was also reported. RMSEA values of 0.05 to 0.08 represent reasonable fit, and values of 0.05 or less indicate good fit [18].

To provide additional information on the psychometric properties of the WDEQ-A items Rasch analysis was conducted on the original WDEQ-A scale, and each of the subscales identified using EFA. These analyses were conducted using Sample 1 (*n* = 683). Drawing on modern test theory Rasch analysis provides much more detailed diagnostic information about the scale, over and above that provided by the traditional classical test theory approaches (EFA and CFA). It is increasingly being used in the social and health sciences to assess scale items, response format, item bias, dimensionality, and targeting (For a detailed description see Pallant and Tennant [41]).

Briefly the aim of Rasch analysis is to test the fit of the data against a mathematical model representing measurement (the Rasch model) developed by Danish mathematician Georg Rasch [42]. Initially overall fit of the model is assessed using two different indicators (a fit residual standard deviation value of 1.4 or less, and a non-significant chi square value). The fit of each of the individual items is further assessed using chi square statistics and individual fit residuals, with values of 2.5 indicating poor fit. The appropriateness of the response scale is checked by inspecting the threshold map for the presence of disordered thresholds. The Person Separation Index (PSI) is used to evaluate the internal consistency reliability of the scale, with values of .70 or above considered acceptable [43]. The dimensionality of the scale is explored by conducting a Principal Components Analysis (PCA) on the residual correlation matrix to identify subsets of items. A series of t-tests are then conducted, comparing Rasch derived scores on these subsets of items, to identify individuals with significantly different scores on the two sets of items. If more than 5% of tests are significant, or the lower bound of the confidence interval exceeds 5%, the scale is considered multidimensional. Local dependency among items is identified by inspection of the residual correlation matrix for values above 0.2.

Using the combined datafile (*n* = 1410) subscale scores were calculated by adding together the items identified using EFA, CFA and Rasch Analysis. Spearman correlation coefficients were calculated among the subscales, and with other scales administered as part of the study (FOBS, EPDS). Scores on each of the subscales were compared for nulliparous vs multiparous women.

## Results

### Demographic characteristics

The socio-demographic and obstetric characteristics of the total sample (*n* = 1410), and the samples used for Exploratory factor analysis (EFA: *n* = 683) and for Confirmatory factor analysis (CFA: *n* = 727), are presented in Table 2. In the total sample the ages ranged from 17 to 51 years with a mean of 29 (SD = 5.5). The majority were in a relationship, with only 97 women (7%) indicating they were single. The sample as a whole was quite well educated, with one half completing a diploma or university qualifications. Almost 30% of the sample were not employed, 1% were on maternity leave, with the remainder of the sample employed in casual, part time or full time positions.

**Table 2:** Participant characteristics

Characteristic	Total sample	EFA and Rasch sample	CFA sample
	*n* = 1410	*n* = 683	*n* = 727
Age (M, SD, Range)	29 (5.5)	29 (5.5)	29 (5.5)
	17 to 51	17 to 51	17 to 44 years
Marital status (n, %)			
Not in relationship	97 (7%)	44 (7%)	53 (7%)
Engaged/Defacto	497 (36%)	252 (38%)	245 (35%)
Married	782 (57%)	373 (56%)	409 (58%)
Education n (%)			
Did not complete high school	275 (20%)	147 (22%)	128 (18%)
Completed high school	427 (30%)	188 (28%)	239 (33%)
Diploma	374 (27%)	178 (26%)	196 (27%)
University degree	330 (23%)	168 (25%)	162 (22%)
Employment status n (%)			
Maternity leave	19 (1%)	14 (2%)	5 (1%)
Not employed	386 (29%)	188 (29%)	198 (28%)
Casual	202 (15%)	99 (15%)	103 (15%)
Part time	235 (17%)	111 (17%)	124 (18%)
Full time	506 (38%)	240 (37%)	266 (38%)
Parity			
Nulliparous	609 (43%)	303 (44%)	306 (42%)
Multiparous	801 (57%)	380 (56%)	421 (58%)
WDEQ			
Mean, SD, Range	49.5 (21.9)	48.4 (21.7)	50.5 (22.1)
	0 to 128	3 to 127	0 to 128
Percentage above 85	68 (5%)	28 (4%)	40 (5.6%)

### Exploratory factor analysis

The suitability of the first randomly selected datafile (*n* = 683) for factor analysis was confirmed with a KMO of .94 and a significant Bartlett’s Test of Sphericity (*p* < .001). Principal components analysis (PCA) of the 33 WDEQ-A items revealed seven factors with eigenvalues above 1, however parallel analysis indicated that only five factors were appropriate for retention. The screeplot suggested either a three or five factor solution, therefore both solutions were inspected.

Oblimin rotation of the 3-factor solution was not optimal, with a number of items failing to load above .45 on any factor and other items showing substantial loadings (above .40) on multiple factors. The five factor solution was interpretable, however to achieve ‘simple structure’, it was necessary to remove one factor containing only two items, and four additional items which failed to load above .40 on any factor or showed crossloadings (20: *hopelessness*; 21:*longing for the child*; 26: *allow my body to take total control*; 31: *dangerous*). The factor that was removed consisted of two items (item 32, item 33) that referred to concerns that the child would die or be injured during labour/birth. These items were strongly intercorrelated (*r* = 0.67), but showed very low correlations with other WDEQ-A items.

The final four-factor solution for the remaining 27 items explained a total of 60.46% of the variance, with all items loading above .45 on their respective factor (see Table 3). The 12 items loading on Factor 1 referred to lack of positive emotions and feelings of confidence, while Factor 2 contained four items relating to social isolation. The third factor contained only three items which are grouped together in the WDEQ-A asking women to imagine how they would feel at the moment of birth. The final factor consisted of eight items representing negative emotions of panic, fear and tension. The items representing each of these factors are presented in Table 3.

**Table 3:** Pattern matrix with oblimin rotation of four-component solution of WDEQ items

	Component			
	Lack of positive emotions	Social isolation	Moment of Birth	Negative emotions
13 glad	**.832**	.062	-.079	-.172
18 happy	**.805**	.057	-.118	-.170
14 proud	**.786** ^a^	.097	-.129	-.229
17 relaxed	**.655**	-.184	.035	.363
22 self confidence	**.627** ^a^	.017	-.071	.230
10 independent	**.587**	-.031	.033	.278
4 strong	**.587** ^a^	.051	.010	.253
5 confident	**.582**	.013	-.051	.288
23 trust	**.569**	.172	-.141	.069
1 fantastic	**.537** ^a^	.081	-.064	.060
16 composed	**.477**	-.051	-.012	.423
9 safe	**.452**	.250	-.120	.087
7 deserted	-.017	**.862**	.028	.024
11 alone	.059	**.847**	.001	.032
15 abandoned	.036	**.772**	-.032	-.040
3 lonely	.050	**.746**	.035	.137
29 natural	.098	-.028	**-.852**	.038
28 enjoyable	.013	-.080	**-.839**	.026
30 totally as it should be	.089	.038	**-.819**	.067
19 panic	.116	.087	-.043	**.698**
25 behave badly	-.168	.063	-.170	**.695** ^a^
6 afraid	.118	.194	-.024	**.626**
27 totally lose control of myself	-.041	.077	-.150	**.620** ^a^
24 pain	.106	-.275	.085	**.616**
2 frightful	.083	.159	-.038	**.612**
12 tense	.357	.037	.099	**.546**
8 weak	.268	.171	.090	**.524**

Note. Major loadings are shown in bold. ^a^indicates items removed in the later Rasch analysis

### Confirmatory factor analysis

Using a separate datafile (Sample 2: *n* = 727) Confirmatory Factor Analysis (CFA) was conducted to test (a) the original unidimensional model, and (b) the four factor model identified using exploratory factor analysis.

The single factor model, containing all 33 WDEQ-A items, showed very poor fit (e.g. CFI = .69), with all fit indices falling well below acceptable standards [44, 45] (see Table 4). These results failed to support the unidimensional structure of the original WDEQ-A.

**Table 4:** Fit statistics from CFA of WDEQ items (*n* = 718)

Model	Chi sq	df	*p*	RMSEA	CFI	TLI
Single factor solution original 33 items	9332.79	495	<.001	.16	.69	.67
Four factors 27 items	2938.47	318	<.001	.11	.90	.89
Four factors 27 items correlated errors ^a^: 5/4, 27/25, 14/13, 14/18	2220.83	314	<.001	.09	.93	.92

*RMSEA* root mean square of approximation (values < .05 indicate close fit, values between .05 and .08 indicate reasonable fit and values above .10 indicate poor fit, [44])

*CFI* comparative fit index (values above .95 are desirable, [45])

*TLI* tucker-lewis index (values above .95 are desirable, [45])

^a^To improve model fit the error terms between these pairs of items were allowed to correlate, reflecting similarity in item content

In comparison, the four factor solution, consisting of the 27 items retained after EFA, recorded much better fit indices (CFI = .90). These were further improved by correlating the errors of four pairs of items (see Table 4). The final solution recorded fit indices indicating adequate fit according to guidelines provided by Byrne (2010) with a CFI of .93, and a TLI of .92.

### Rasch analysis

Rasch analysis was undertaken to assess (a) the original 33-item WDEQ-A scale and (b) each of the four subscales identified from the previous EFA. Sample 1 (*n* = 683) was used for these analyses.

#### Original WDEQ-A scale

Rasch analysis of original 33 item version of the WDEQ-A showed serious deviations from the Rasch model. Both the chi-square probability value (*p* < .0001) and fit residual standard deviation value (4.61) greatly exceeded the recommended guidelines, suggesting misfit among the items (see Table 5). Dimensionality testing failed to support the unidimensionality of the scale, with 23% of the sample recording statistically significant different scores on the sets of items tested (see Table 5: Analysis 1). This is well above the acceptable level of 5%. Ten of the items recorded disordered thresholds, indicating problems with the response format of the scale. These results clearly do not support the addition of all 33 items to form a total score.

**Table 5:** Summary of Rasch analysis on WDEQ subscales

Subscale	Analysis	Overall model fit	Item fit residual mean (SD)	Person fit residual mean (SD)	PSI	% sig t-tests^a^
All 33 WDEQ items	1	Chisq = 1377.67, df = 297, *p* < .0001	1.4 (4.61)	-0.81 (1.77)	.95	23.3% CI:21.7–24.9
Lack of positive emotions (12 items)	2	Chisq = 164.94, df = 108, *p* < .0001	.41 (2.22)	-.44 (1.43)	.92	9.09% CI:7.5–10.7
Lack of positive emotions (8 items) Remove items 1 22 4 14	3	Chisq = 88.43, df = 72, *p* = .09	.66 (.98)	-.43 (1.30)	.89	9.97% CI: 8.3–11.6
Lack of positive emotions (5 items) Remove items 1 22 4 14 13 10 16	4	Chi sq= 74.16, df = 48, *p* = .004	.26 (1.05)	-.38 (1.06)	.82	4.84%^a^
Negative emotions (8 items)	5	Chisq = 198.75, df = 71, *p* < .0001	.46 (3.31)	-3.8 (1.31)	.85	10.4% CI: 8.8–12.0
Negative emotions (5 items) Remove items 24 25 27	6	Chisq = 63.38, df = 45, *p* = .04	.20 (1.93)	-.59 (1.38)	.84	5.28% CI: 3.6–6.9
Moment of birth (3 items)	7	Chisq = 45.42, df = 15, *p* < .0001	.14 (1.30)	-.66 (1.19)	.59	b
Social isolation (4 items)	8	Chisq = 42.91, df = 20. *p* = .002	.11 (1.20)	-.55 (1.19)	.43	b

*Chisq* Chi square, *CI* confidence interval, *df* degrees of freedom, *p* probability, *PSI* Person Separation Index, *SD* standard deviation

^a^confidence interval only reportedx if the % value exceeds 5%

^b^unidimensionality testing not conducted due to small number of items

Note. Overall fit to the model is indicated by a chi square *p* value that is not significant, Item fit residual SD values and Person ft residual SD values should be less than 1.5, and the percentage of significant t = tests shold not exceed 5%

#### WDEQ-A: Lack of positive emotions subscale

Rasch analysis was also undertaken on the four subscales identified from the previous EFA. Initially the 12 items of the *Lack of Positive Emotions* subscale showed some misfit to the model, with a chi square probability value of *p* < .0001, and an item fit residual SD value of 2.22 (see Table 5, Analysis 2).

To achieve acceptable fit it was necessary to remove four items (items 1, 22, 4, 14). This solution showed good fit to the Rasch model, with a non-significant chi square probability value of .09, and a fit residual SD of .98 (see Table 5: Analysis 3). The scale had good internal consistency reliability (PSI = .89) and no disordered thresholds. There was no DIF for age or education level and no local dependency among the items. As shown in Table 5 (Analysis 3) over 9% of the sample recorded statistically significant differences in scores on the subtests used for dimensionality testing, exceeding the recommended level of 5%. It was decided to remove a further three items (10, 13, 16) to resolve this multidimensionality issue and to further shorten the scale for ease of use. The final solution (Analysis 4) consisted of five items which showed no misfitting items, good internal consistency and no evidence of DIF, local dependency or multidimensionality.

#### WDEQ-A: negative emotions subscale

Initial Rasch analysis of the eight *Negative Emotions* items suggested the presence of misfitting items and multidimensionality (see Table 5: Analysis 5). Both these issues were resolved by the removal of three items (24, 25, 27). The final five-item subscale showed good internal consistency reliability (PSI = .84), no misfitting items, no DIF for age or education, and no local dependency among the items. The subscale met the requirements for unidimensionality with the lower bound of the confidence interval falling below 5% (see Table 5: Analysis 6).

#### WDEQ-A: moment of birth subscale

The three-item *Moment of birth* subscale showed adequate fit to the model (fit residual SD = 1.30) with no misfitting items, no disordered thresholds, and no local dependency (see Table 5: Analysis 7). Comparison of the PSI value (.59) and the Cronbach alpha value (.84) revealed a substantial difference, suggesting a very skewed distribution of scores. This was confirmed by an inspection of the item-threshold map showing a substantial proportion of the sample (*n* = 262, 38%) recording very low scores on the scale.

#### WDEQ-A: social isolation subscale

Rasch analysis of the four-item Social Isolation subscale showed fit to the Rasch model with a satisfactory item fit residual SD (1.20), no misfitting items, no DIF for age or education, and no local dependency (see Table 5: Analysis 8). Two items (7 and 15) recorded minor disordering of the response thresholds. Scores on this scale were very skewed, with 355 respondents (52%) recording extreme scores, responding with a 0 for all items. This is also reflected in the marked discrepancy between the PSI value (.43) and the Cronbach alpha value (.85).

### Descriptive statistics and subscale intercorrelations

Total scores were calculated for each of the four subscales of the WDEQ-A-Revised using only those items that were retained following EFA, CFA and Rasch analysis. The total scores were divided by the number of items in each subscale, resulting in scores ranging from a possible 0 to 5. All subscales showed good internal consistency reliability (see Cronbach alpha values and descriptive statistics in Table 6). Percentile values (25^th^, 50^th^, 75^th^) are included to provide comparison values for future users of these subscales. The number and percent of cases exceeding the midpoint of the subscale (2.5) are provided as a suggested cutpoint for identifying women who may benefit from further investigation.

**Table 6:** Descriptive statistics for WDEQ-Revised subscales and correlations with other measures

	WDEQ-Revised subscale			
	Negative emotions	Lack of positive emotions	Social isolation	Moment of birth
	*N* = 1404	*N* = 1406	*N* = 1405	*N* = 1405
Number of items	5 items	5 items	4 items	3 items
25^th^ percentile^a^	1.2	1.20	0	0
50^th^ percentile^a^ (median)	1.80	1.80	0.0	.67
75^th^ percentile^a^	2.4	2.4	1.0	1.3
Range^b^	0–5	0–5	0–4	0–5
n (%) of cases exceeding the cutpoint of 2.5	302 (21.5%)	417 (24.6%)	54 (3.8%)	122 (8.7%)
Cronbach Alpha	.87	.82	.86	.86
Intercorrelations among WDEQ subscales^cd^				
Negative emotions	-			
Lack of Positive emotions	.66	-		
Social isolation	.39	.45	-	
Moment of birth	.29	.49	.34	-
Correlations with other measures^d^				
FOBS-The Fear of Birth Scale^TM^	.65	.58	.31	.29
EPDS-Total	.38	.37	.33	.23
EPDS-3A	.34	.33	.27	.20

^a^Note. Scores on each WDEQ-Revised subscale are calculated by adding the score for each item (0 to 5) and dividing by the number of items in the subscale. This allows comparison across subscales with different number of items

^b^Possible score range 0 to 5

^c^All WDEQ subscale intercorrelations are significant at *p* < .001

^d^Spearman correlation coefficient

There was a marked difference in the distribution of scores on the various subscales. Both the *Social Isolation* and *Moment of Birth* subscales were extremely skewed, with a substantial proportion of the sample recording a score of 0 (52% for Social Isolation, and 38% for Moment of Birth). The *Positive Emotions* and *Negative Emotions* subscales however showed a good spread of scores across the range of possible values.

Table 6 shows the intercorrelations among the four WDEQ-A-Revised subscales. The strongest correlation was between the *Lack of Positive Emotions* and *Negative Emotions* subscale (*r* = .66) suggesting 44% shared variance, while the lowest was between the *Moment of Birth* and *Negative Emotions* (*r* = .29) indicating only 8% shared variance. This very low association between subscales was further explored by dividing each subscale score at the median to create two groups (low/high). Forty percent of women (*n* = 560) recorded inconsistent classification on these two subscales (low on one subscale, high on the other) suggesting that the scales are tapping different characteristics.

Correlations between the WDEQ-A-Revised subscales with other measures were also calculated to assess the degree to which the subscales are measuring the same underlying characteristic (see Table 6). If the WDEQ-A is unidimensional, as proposed by the original authors, then the subscales should show a consistent pattern of association with other scales. The results shown in Table 6 indicate that this is not the case. Although the correlations between the *Negative emotions* and *Lack of Positive Emotions* with FOBS-The Fear of Birth Scale^TM^ [26] were strong as expected (*r* = .65 and *r* = .58 respectively), the correlations recorded with the other two WDEQ-A-R subscales were much weaker (*Social Isolation* scale *r* = .31, *Moment of Birth r* = .29). This suggests that these latter two subscales are not measuring fear of birth. These two subscales also recorded lower correlations with the Edinburgh Postnatal Depression Scale when compared with the corresponding correlations recorded for the *Negative emotions*, and *Lack of positive emotions* (see Table 6).

Independent groups t-tests indicate that scores on all four WDEQ-A-Revised subscales were significantly higher for the nulliparous women (see Table 7), however the effect size for the *Negative Emotions* was much stronger (partial eta squared = .10), than either the *Social Isolation* or *Moment of Birth* subscales (both partial eta squared = .006).

**Table 7:** Comparison of WDEQ subscale scores for nulliparous and multiparous women

	Negative emotions	Lack of positive emotions	Social isolation	Moment of birth
Parity				
Nulliparous (*n* = 606) M(SD)	2.19 (.93)	1.97 (.86)	.58 (.80)	.97 (.99)
Multiparous (*n* = 798) M(SD)	1.58 (.90)	1.69 (.92)	.46 (.72)	.82 (.97)
Independent t tests: t(df)	12.38 (1402)	5.94 (1346)	2.81 (1403)	2.86 (1403)
* p*	<.001	<.001	0.005	0.004
Effect size (partial eta squared)	0.10	0.025	0.006	0.006

The final WDEQ-A Revised can be seen in Additional file 1.

## Discussion

The extensive psychometric evaluation of the WDEQ-A undertaken in this study clearly suggests that it is multidimensional and that it is not appropriate to calculate a total score. In this large sample of 1410 Australian women, four separate subscales were identified (*Negative emotions, Lack of positive emotions, Social isolation and Moment of birth*), each showing good internal consistency. The pattern of correlations among the WDEQ-A-Revised subscales, and their correlation with other measures (FOBS-The Fear of Birth Scale^TM^, EPDS), also suggest that the subscales are measuring different aspects of the underlying concept and therefore should not be combined.

The results of this study are consistent with a growing number of other studies drawing attention to the lack of unidimensionality of the WDEQ-A [9, 10, 14]. The four subscales obtained in the current study are similar, but not identical, to those obtained in other factor analytic studies of the WDEQ-A [9, 10, 14, 15]. The 5-item *Negative emotions* subscale in the current study was similar in content to a factor labeled *Fear* by other authors [9, 14]. Our subscale *Social Isolation* was consistent with items labeled *Isolation* by Fenwick et al. [14] and *Loneliness* by Lukasse et al. and Garthus-Niegal et al. [12, 16]. Five of the items in our *Lack of positive emotions* subscales were also present in the factor labeled by Fenwick et al. [14] as *Lack of positive anticipation*. Unlike previous researchers we identified a fourth subscale which contains items from the section in the WDEQ-A which asked women “How do you imagine it will feel the very moment you deliver the baby?” This subscale was therefore labeled *Moment of birth* to distinguish it from the remaining items in the WDEQ-A which asked women to think about how they would feel during the labour and delivery*.*

This study utilized techniques from both classical test theory (exploratory and confirmatory factor analysis) and modern test theory (Rasch analysis) to fully explore the psychometric properties of the WDEQ-A. This study was the first to utilize Rasch analysis, one of the family of a modern test theory techniques that is now widely being used to explore all aspects of a scale’s performance. It involves assessment of the fit of each of the items, the response scale, item bias, and dimensionality, and allows identification of weak items for removal from the scale. After removal of items based on the application of Rasch analysis, the final 17 -item four-subscale version of the WDEQ-A (referred to as WDEQ-A-Revised) showed good psychometric properties. The removal of poorly performing or unnecessary items resulted in a shorter, more concise measure, better suited to research and clinical utilisation.

Although previous researchers have reported concerns about the multidimensionality of the WDEQ-A, many have continued to calculate a total WDEQ-A score and to use cutpoints based on this score [12, 46]. To derive a total score for a scale it is essential that the items included in the scale are all measuring the same underlying construct [47]. The low intercorrelations among some of the subscales in this study clearly suggest that they are tapping different domains and therefore should not be combined. This is particularly evident for the *Social isolation* and *Moment of Birth* subscales which both showed correlations of less than .40 with other subscales. A correlation of .40 represents only 16% overlap in the subscales [32], indicating that respondents with high scores on one subscale, do not necessarily record high scores on other subscales. The combination of subscales with low intercorrelations to create a total score is meaningless.

If the WDEQ-A is not unidimensional, as suggested by this, and a growing number of other studies worldwide, a question is raised concerning its research and clinical utility. How can it be used to assess the level of fear experienced by women facing childbirth? One option is to explore the unique contribution of each of the WDEQ-A-Revised subscales separately. The *Negative emotions* subscale identified in the current study most closely represents fear of childbirth, containing the items *panic, afraid, tense, frightful and weak*. Women who consistently endorse above the midpoint on each of these items (representing a mean score of 2.5 or above) may warrant further investigation concerning their perceptions of the upcoming birth. In this study that represents 21.5% of the sample. Elevated scores on the *Social Isolation* subscale may also be of clinical concern. In our study only 3.8% (*n* = 54) had a mean score of 2.5 or above on this subscale. Women who feel *deserted, alone, lonely or abandoned* are potentially at increased risk when facing the challenges of childbirth and motherhood, and may require additional support services, during both the birth and the postpartum period. It is possible that the positive emotions indicated by low scores on the *Lack of positive emotions* subscale (e.g. *confident, relaxed, happy, composed, safe*) may serve as a buffer, moderating any potential impact of elevated fear levels. Using the scores from the profile of scores on the *Negative emotions, Lack of positive emotions* and *Social isolation* subscales may prove potentially useful in the identification of women requiring counseling, giving top priority to those women with high fear levels that also have a lack of protective positive emotions, and perceived lack of support.

It is unclear how the scores on the *Moment of birth* subscale could contribute as a either a research or clinical tool, particularly for women who have not yet experienced the birth process. Unlike other items in the scale, which address feelings during both labour and delivery, these items refer specifically to how the woman imagines she will feel the very moment of delivery. This scale recorded the lowest intercorrelation values, both with other WDEQ-A subscales, and other validated tools (EPDS and FOBS^TM^). A number of the items in this subscale (eg. Q28 funny, Q30 self-evident) have been identified as difficult for respondents to understand, perhaps due to difficulties in translating from the original Swedish. In a number of previous studies the wording of these items have been modified differently across studies see for example Johnson and Slade and Fenwick et al. [10, 14], making it difficult to compare.

Further investigation of the WDEQ-A-Revised is required to fully understand how each individual subscale may contribute to a better understanding of the emotional health of women facing childbirth. This study was conducted on a large cohort of Australian women, using an English version of the WDEQ-A. Given the widespread use of the scale, further validation of this revised 17 -item, 4-subscale version (WDEQ-A-Revised) is required in different cultural groups. This study focused on the internal validity of the scale and included a very limited range of other measures suitable for exploring the external validation of the tool. Additional studies exploring the association between the WDEQ-A-Revised subscales with other validated tools is needed, and longitudinal studies are required to explore the impact on the birth process and outcome. These investigations will help to determine the unique contribution, and potential usefulness, of the four individual subscales. Additional investigation of the WDEQ-A-Revised is also needed to determine its suitability to clinical practice, particularly given the availability now of shorter, easy to administer, tools such as the FOBS- The Fear of Birth Scale^TM^ which may be more appropriate in time pressured settings such as hospitals and clinics.

New cutpoints for the WDEQ-A-Revised will need to be established to guide clinicians and researchers in the identification of what constitutes high levels of childbirth fear. The score of >2.5 would suggest that a woman has chosen a response option above the midpoint of the scale on each of the items. To allow comparison of the levels of childbirth fear in future studies the percentage of this sample recording above the midpoint are provided in Table 6. Pending further clinical investigation of this issue it is recommended that women recording above the midpoint (2.5) on each subscale should be considered for further investigation. This could be in the form of further sensitive questioning by the midwife or doctor caring for the woman to understand the content of her fears. By using good clinical judgment, possibly aided by additional screening for anxiety and depression, the woman may be referred to the appropriate resource for more detailed assessment.

## Conclusion

The results of this study support the findings of a growing number of other studies that suggest that the WDEQ-A is multidimensional and therefore a total scale score should not be calculated. The four subscales identified in the current study may provide researchers with a more refined, psychometrically sound tool to explore the concept of fear of birth, and the differential impact of the various aspects included in the WDEQ-A-Revised. Further testing is needed to assess the revised format of the WDEQ-A in different cultural settings, and to evaluate its potential applicability in clinical settings to evaluate the emotional health of women facing childbirth and identify those at risk.

## Additional file

Additional file 1: WDEQ-A Revised: The 17 item WDEQ-A Revised with four subscales. (DOCX 13 kb)

## Abbreviations

CFA: Confirmatory factor analysis
CFI: Comparative fit index
CI: Confidence interval
DIF: Differential item functioning
EFA: Exploratory factor analysis
EPDS: Edinburgh post natal depression scale
FOBS: Fear of birth scale
GFI: Goodness of fit index
KMO: Kaiser-Meyer-Olkin
PCA: Principal components analysis
RMSEA: The root mean-square error of approximation
WDEQ-A: Wijma delivery expectancy questionnaire
