# Supplementary material for: Assessment of the dimensionality of the Wijma delivery expectancy/experience questionnaire using factor analysis and Rasch analysis
Source: BMC Pregnancy Childbirth. 2016 Nov 21;16:361. doi: 10.1186/s12884-016-1157-8 (PMC5117613; doi:10.1186/s12884-016-1157-8)
Supplement: Additional file 1: — WDEQ-A Revised: The 17 item WDEQ-A Revised with four subscales. (DOCX 13 kb) [file 12884_2016_1157_MOESM1_ESM.docx]

| Item  Number | **List of 17 items, 4-subscale version of WDEQ-A*** |
| --- | --- |
| **Negative emotions** | |
| 2 | **How do you think your labour and delivery will turn out as a whole?**  0= Extremely frightful 5= Not at all frightful (reverse scored) |
| 6 | **How do you think you will feel in general during the labour and delivery?**  0= Extremely afraid 5=Not all afraid (reverse scored) |
| 8 | **How do you think you will feel in general during the labour and delivery?**  0=Extremely weak 5=Not at all weak (reverse scored) |
| 12 | **How do you think you will feel in general during the labour and delivery?**  0= Extremely tense 5=Not at all tense (reverse scored) |
| 19 | **How do you think you will feel during labour and delivery?**  0= Extreme panic 5= No panic at all (reverse scored) |
| **Lack of Positive emotions** | |
| 5 | **How do you think you will feel in general during the labour and delivery?**  0= Extremely confident 5= Not at all confident |
| 9 | **How do you think you will feel in general during the labour and delivery?**  0= Extremely safe 5= Not at all safe |
| 17 | **How do you think you will feel in general during the labour and delivery?**  0= Extremely relaxed 5= Not at all relaxed |
| 18 | **How do you think you will feel in general during the labour and delivery?**  0=Extremely happy 5= Not at all happy |
| 23 | **How do you think you will feel during the labour and delivery?**    0= Extreme trust 5= No trust at all |
| **Social isolation** | |
| 3 | **How do you think you will feel in general during the labour and delivery?**  0= Extremely lonely 5= Not at all lonely (reverse scored) |
| 7 | **How do you think you will feel in general during the labour and delivery?**  0= Extremely deserted 5= Not at all deserted (reverse scored) |
| 11 | **How do you think you will feel in general during the labour and delivery?**  0= Extremely alone 5= Not at all alone (reverse scored) |
| 15 | **How do you think you will feel in general during the labour and delivery?**  0= Extremely abandoned 5= Not at all abandoned (reverse scored) |
| **Moment of birth** | |
| 28 | **How do you imagine it will feel the very moment you deliver the baby?**  0= Extremely enjoyable 5= Not all enjoyable |
| 29 | **How do you imagine it will feel the very moment you deliver the baby?**  0= Extremely natural 5= Not at all enjoyable |
| 30 | **How do you imagine it will feel the very moment you deliver the baby?**  0= Totally as it should be 5=Not at all as it should be |

*Note: This table describes the four subscales and items contained within each. It is not intended for use as a questionnaire in this format.
